# Editorial: Inflammation in the female genital tract

**DOI:** 10.3389/frph.2023.1161839

**Published:** 2023-03-09

**Authors:** Alicia Berard, Julie Lajoie, Carolina Herrera

**Affiliations:** ^1^ Department of Obstetrics & Gynecology, University of Manitoba, Winnipeg, MB, Canada; ^2^ Medical Microbiology and Infectious Diseases Department, University of Manito, Winnipeg, MB, Canada; ^3^ Department of Infectious Diseases, Imperial College London, London, United Kingdom

**Keywords:** inflammation, reproductive health, cytokines, immune response, sexually tranmistted infections, microbiome

**Editorial on the Research Topic** Inflammation in the female genital tract

A global call for action to become more inclusive in sex and gender differences in biological sciences has highlighted the need for more studies focused on women's health, including research in reproductive health. This special issue in Frontiers Reproductive Health is bringing forward some of the most current studies on how inflammation plays a role in women's reproductive health to highlight its impact on the female genital tract.

Women's reproductive health is largely linked with inflammatory events that can occur during a woman's lifetime. Normal reproductive function involves strict regulation of the inflammatory pathways in order to re-establish homeostasis dysregulated after sexual intercourse, birth or repair from diseases and infection. Recent literature has indicated that without proper resolution, the maintenance of a genital inflammatory state is a key factor in numerous pathologies such as pelvic inflammatory diseases and cancers, but it also increases the risk of acquiring sexual transmitted infections (STIs) or experiencing pre-term birth (Adapen et al.). Inflammation can come from many sources such as presence of an STI, changes in the vaginal microbiome, changes in hormones levels due to age or other factors, pregnancy and labour, cancers, uses of some hormonal contraceptives and sexual activities (Adapen et al.; Gholiof et al.).

Microbicides and other preventive compounds used in clinical trial to prevent STIs have showed that genital inflammation plays an important role for product effectiveness. The nonoxynol-9 microbicide, for example, failed to prevent HIV and increased the risk of acquiring HIV and other STIs. This microbicide was later associated with increase genital inflammation (1). More recently, the Caprisa 004 study showed that while the use of this gel could reduce the risk of HIV infection by 39% (2), an increased baseline genital inflammatory state was associated with negating this protective effect (3). To determine the presence of inflammation in the genital tract, the measurement of cytokines previously associated with risk of STI acquisition may be used as a guideline for evaluating novel vaginal products in clinical trials, as is evaluated in the review by Happel et al.

Inflammation of the vaginal mucosal environment can be influenced by the resident vaginal microbiome, with a dysbiotic microbiome inducing an increase in inflammation. The role of the microbiome in female reproductive health is currently being investigated with numerous clinical and *in vitro* studies, however much is still not well characterized. Adapen et al. has reviewed our current knowledge on cytokine profiles, immune cell recruitment and their interactions with the microbiome that influence local inflammation. A reliable method of investigating the vaginal microbiome in an animal model is greatly needed to further mechanistic studies of female reproductive tract health related to the microbiome. Adapen et al. have also reviewed the effectiveness of utilizing a non-human primate model to evaluate the microbiome in the context of regulating inflammation (Adapen et al.).

Two articles in this issue touch on the role of neutrophils in the female genital tract. The microbiome has been shown to both influence and be influenced by neutrophils in the vaginal mucosa (Adapen et al.). A novel study by Cheu et al. used endocervical cytobrushes collected from women with or without BV to assess the bacterial community types as well as the frequency of the neutrophils. They demonstrated that there was an increase in neutrophil frequency, activation, and lifespan in women with a dysbiotic microbiome. Performing *in vitro* analysis Cheu et al. also showed that dysbiotic bacteria strains can induced neutrophil activation and increased lifespan directly (Cheu et al.; Tyssen et al.).

Treatment of a dysbiotic microbiome, such as during bacterial vaginosis, using over the counter vaginal products is common practice for women. However, these products need to be studied to determine whether they are safe to use without causing more local inflammation. Unfortunately, the impact on the vaginal barrier and inflammation of many of these gels has not been comprehensively studied (Tyssen et al.). Happel et al. have reviewed what is needed to take into consideration when performing safety assessments during pre-clinical trials for vaginal products. These guidelines include how best to design the study by collecting numerous baseline samples, assessing the product over time, and determining the most dilute concentration of product that has the desirable effect. In addition, they have identified key cytokine biomarkers based on literature findings to evaluate safety of these products.

The assessment of available vaginal gels on the epithelial barrier and cytokine expression was performed by Tyssen et al. in this issue. They focused their study on gels that contain lactic acid, as this is an antibacterial and antiviral agent, measuring osmolality of these gels plus a few others without lactic acid, and determine using *in vitro* assays whether these gels damage barrier integrity measured using transepithelial electrical resistance and histology of EpiVaginal tissue models. They determined that hyperosmolal gels detrimentally affected electrical resistance, and most gels induced moderate tissue damage. This indicates the need for better evaluation of vaginal products prior to being used, by measuring not only the inflammation but also the epithelial barrier integrity (Tyssen et al.).

In summary, though many studies are now focusing on women's health, there is still a lot that needs to be done to better understand the environmental risks that are associated with poor reproductive health and acquisition of STIs. As a community, we need to characterize the natural immune environment of the female genital tract. In addition, we need to understand how vaginal products or exogenous hormones can lead to genital inflammation and epithelial damage prior to being made available for use in order to prevent any increase in risk associated with genital inflammation.
